# Exploring the function of stromal cells in cholangiocarcinoma by three-dimensional bioprinting immune microenvironment model

**DOI:** 10.3389/fimmu.2022.941289

**Published:** 2022-08-02

**Authors:** Changcan Li, Bao Jin, Hang Sun, Yunchao Wang, Haitao Zhao, Xinting Sang, Huayu Yang, Yilei Mao

**Affiliations:** Department of Liver Surgery, Peking Union Medical College (PUMC) Hospital, PUMC & Chinese Academy of Medical Sciences (CAMS), Beijing, China

**Keywords:** 3D bioprinting, immune microenvironment, cholangiocarcinoma (CCA), tumor-associated fibroblasts, tumor-associated endothelial cells, tumor-associated macrophages

## Abstract

The tumor immune microenvironment significantly affects tumor progression, metastasis, and clinical therapy. Its basic cell components include tumor-associated endothelial cells, fibroblasts, and macrophages, all of which constitute the tumor stroma and microvascular network. However, the functions of tumor stromal cells have not yet been fully elucidated. The three-dimensional (3D) model created by 3D bioprinting is an efficient way to illustrate cellular interactions *in vitro*. However, 3D bioprinted model has not been used to explore the effects of stromal cells on cholangiocarcinoma cells. In this study, we fabricated 3D bioprinted models with tumor cells and stromal cells. Compared with cells cultured in two-dimensional (2D) environment, cells in 3D bioprinted models exhibited better proliferation, higher expression of tumor-related genes, and drug resistance. The existence of stromal cells promoted tumor cell activity in 3D models. Our study shows that 3D bioprinting of an immune microenvironment is an effective way to study the effects of stromal cells on cholangiocarcinoma cells.

## Introduction

Cholangiocarcinoma (CCA) is the most common biliary malignancy and the second most common primary liver malignant tumor, only after hepatocellular carcinoma (1, 2). CCA accounts for 10–20% of deaths due to hepatobiliary malignancies (3, 4). Usually, early diagnosis of CCA is challenging, as it has no specific symptoms; thus, it is mostly diagnosed at an advanced stage. Even if patients undergo standard surgical interventions, they may face a high rate of recurrence and distant metastasis. This leads to poor prognosis, high mortality rates, and limited treatment options; less than 40% of patients survive for more than five years (5, 6). Patients with unresectable CCA generally have a survival rate shorter than 12 months after diagnosis, and neither radiation nor standard-of-care chemotherapy regimens (gemcitabine, 5-fluorouracil, and cisplatin) exhibit significantly improved survival rates (7–10).

A typical histological feature of CCA is desmoplasia, which is the presence of abundant fibrotic stroma that surrounds and infiltrates the tumor structures and a rich tumor immune microenvironment (11). The tumor stroma is so prominent that it outweighs the tumoral component (12). Stroma contains both non-immune and immune cell types, as well as capillary networks, including tumor-associated fibroblasts (TAF), tumor-associated endothelial cells (TEC), and lymphatic cells, such as tumor-associated macrophages (TAM), tumor-associated neutrophils, and regulatory T lymphocytes (Tregs). These cells affect CCA progression through various mechanisms, such as migration, invasion, metastasis, immune responses, angiogenesis, and lymphangiogenesis (11).

A traditional two-dimensional (2D) cell culture model enables adequate nutrient exchange and cell growth, but the cells also lose many inherent characteristics that they possess in three-dimensional (3D) environment *in vivo*, such as a 3D growth environment, intercellular junctions, and cell-matrix interactions (13, 14). Cells have shown non-negligible differences between 2D and 3D models in terms of gene and protein expression, signal transduction, cell migration, cell morphology, proliferation characteristics, and viability (15–17). Hence, 2D models fails to recapitulate the natural microenvironment in tissues or organs and may provide misleading results, especially during the investigation of the pathological mechanism of cancer and anti-cancer drug testing/development (18, 19).

Many 3D cancer models have been designed to overcome the limitations of the current cancer models and reduce the costs of studies and preclinical drug evaluation. Patient-derived xenografts (PDXs) and organoids are the most commonly used 3D cancer models, which have contributed significantly to cancer research (20). These models can better mimic original tissue features in terms of structural organization, cell-cell interactions, and cell–extracellular matrix (ECM) interactions. However, there are also some problems with PDXs, such as ethical disputes, more time consumption, high cost, and complicated operation (21–24). In addition, PDXs models require the use of immunocompromised animals, which lack a fully functional immune system; therefore, they cannot be used to test immunotherapy. In addition, xenograft tumors grow faster than human tumors because of the lack of an immune system. Hence, immature blood vessels inside xenograft tumors do not correspond to tumorigenic blood vessels inside human tumors, and the therapeutic efficacy of compounds exhibited in animal experiments is different from that in humans (25). Organoids can better maintain the characteristics of primary and tumor cells than PDXs in long-term cultures (26). Although organoids have many advantages over traditional models and *in vitro* models of tumors, they are unable to completely replicate the complexity and diversity of primary cells and lack elements of the immune system and vascular factors, especially key stromal cells (27). Therefore, the development of novel 3D tumor tissues that can be used as tumor models for pre-clinical studies is highly desirable (28, 29).

To overcome these limitations, in recent years, advancement of 3D bioprinting technology inspired bioengineers and scientists to develop approaches to “print” *in vitro* tumor-mimicking models to investigate the biological mechanism of tumor development (30). 3D bioprinted model can achieve an accurate and controllable distribution of cells, active molecules, and biomaterials with a complex structure of multi-cell and multi-material arrangements. This technology allows for precise 3D construction of complex tissues and organs (31, 32). Research has shown that co-culture of tumor cells with tumor immune microenvironment-associated cells is a novel approach for which has aided in discovering various novel aspects of the tumor immune microenvironment (33). The biomimetic environment of the 3D bioprinted model recapitulates numerous features of the natural ECM, such as biophysical and biochemical cues, which are essential for cell behavior and growth (34–36). Compared to traditional cell cultures, 3D bioprinted models can provide intercellular junctions and immune microenvironments that closely resemble in the tumor. 3D bioprinting has been considered a suitable technology and applied for the construction of *in vitro* tumor tissue models with a biomimetic tumor microenvironment for pathological studies and drug evaluation (37).

**Figure 1:**
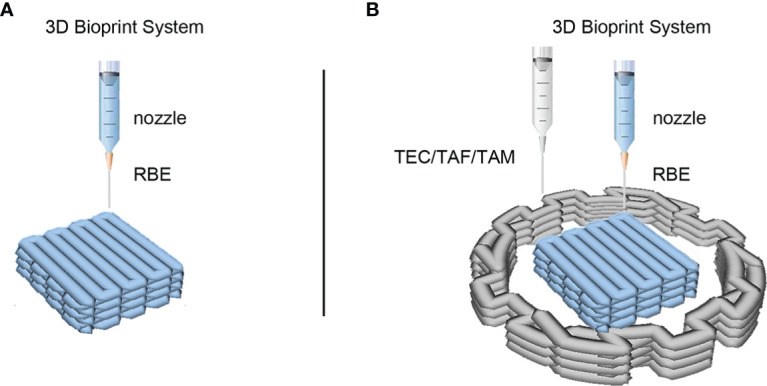
3D bioprinted models of cholangiocarcinoma (CCA) immune microenvironment. **(A)** The schematic of 3D bioprinted model of CCA with RBE alone. **(B)** The schematic of 3D bioprinted model with CCA cells RBE and stromal cells. The middle square structure in blue represents hydrogel containing RBE cells, while the peripheral structure in grey represents hydrogel containing TEC, TAF, or TAM cells according to different models.

In the present study, we established a CCA model using 3D bioprinting technology. Direct mixing of tumor and stromal cells in a 3D environment will cause contact inhibition of both types of cells during growth (38). Therefore, we used a bioprinter platform to print structures composed of cancer cells surrounded by stromal cells (**Figure 1**). First, we confirmed that cells in the 3D bioprinting system had better viability and metabolic activity than those in 2D culture. We then observed the effects of stromal cells on the proliferation, invasion, metastasis, stemness, and drug resistance of CCA cells using a 3D bioprinted model. The 3D bioprinted tumor model displayed a physiological state similar to that found *in vivo* and was compatible with the continuous monitoring and functional evaluation of long-term culture. However, Sun et al. reported that they used isolated primary CCA cells to fabricate a 3D bioprinted model *in vitro* (39). Active proliferation and biological functions of the cells were observed, as well as higher drug resistance was verified successfully. The study focused on the role of CCA cells, as the isolated cells were mainly CCA cells, and the primary medium had screening effects on tumor cells; hence, the stromal cells were absent in the model, which had a non-negligible impact on tumor cells. Therefore, the response of stromal cells to CCA cells could not be assessed in this model, and the model cannot be used for the exploration of the tumor immune microenvironment. In general, our 3D bioprinted tumor model can be exploited to better emulate the clinical and laboratory scenarios of various cancer types, potentially serve as a powerful clinically accurate platform for preclinical research and drug testing and provide a suitable alternative to animal models.

## Materials and methods

### Cell culture

CCA cell line RBE, human umbilical vein endothelial cells (HUVEC), fibroblasts (CCC-HPF-1) and human monocyte leukemia THP-1 cells were obtained from the Cell Bank of the Type Culture Collection of the Chinese Academy of Sciences (Beijing, China). All cells were cultured in high-glucose Dulbecco’s modified minimum essential medium (H-DMEM; Gibco, Waltham, MA, USA) supplemented with 10% fetal bovine serum (FBS, Gibco), 1% penicillin, and streptomycin (Gibco). The cells were cultured in an incubator at 37°C with 5% CO_2_.

THP-1 cells were differentiated to macrophages using 150 nM phorbol 12-myristate 13-acetate (PMA, Sigma-Aldrich, St. Louis, MO, USA) for 24 h. Then M2 macrophages were obtained by removing the medium and incubating the cells in the medium with 20 ng/mL interleukin-4 (IL-4, R&D Systems, Minneapolis, MN, USA) and 20 ng/mL interleukin-13 (IL-13, R&D Systems) for 72 h. M2 macrophages were then used for 3D bioprinting.

### Cell activation

The cells were activated as per a previously established protocol (40). HUVEC, fibroblasts and M2 stromal cells were activated and transformed into TEC, TAF, and TAM, respectively. Briefly, when RBE were 70% confluent, they were incubated in fresh medium for 24 h. The supernatant was collected and filtered through a 0.22μm filter (Merck Millipore, Billerica, MA, USA) and designated as the RBE-conditional medium. After washing with phosphate-buffered saline (PBS, Solarbio, Beijing, China) gently, HUVEC, fibroblasts and M2 were incubated with RBE-conditioned medium for 72 h and then activated to the TEC, TAF and TAM.

### Construction of the 3D bioprinted model

3D cell bioprinter BIOMARKER (SUNP Biotech, Beijing, China) was used to fabricate the *in vitro* cell model following a previously reported protocol (41, 42). Briefly, RBE and/or other stromal cells (TEC, TAF, and TAM) cultured in 2D environment were harvested and prepared as a suspension in culture medium. The cell suspension, 12.5% gelatin methacryloyl (GelMA) hydrogels (SUNP Biotech), and photoinitiator, 2.5% lithium phenyl-2,4,6 trimethylbenzoylphosphinate (LAP, SUNP Biotech) were mixed at a volume ratio of 1:8:1. The final cell density was 5 × 10^6^/mL. The cell/hydrogel mixture was drawn into a sterilized syringe with a 23G needle and placed in the 3D bioprinter at a controlled temperature. The temperature of the nozzle and forming chamber was 23°C and 8°C, respectively. The models were then fabricated by forced extrusion at a speed of 1.5 mm^3^/s (**Figure 1**), followed by exposure to a blue laser (wavelength is 405 nm) for 20 s to solidify the GelMA, and 3 mL of fully supplemented H-DMEM was added to the dish. The medium was changed every 2 days. The RBE printed alone was named RBE(3D), RBE printed with TEC was named RBE(TEC), RBE printed with TAF was named RBE(TAF), and RBE printed with TAM was named RBE(TAM).

### Cell survival

Cell survival in the 3D bioprinted models was evaluated on days 1, 3, 6, 10, and 15 after bioprinting to assess the viability of cells in GelMA hydrogels. A fluorescent live/dead assay was performed to determine cell survival. Briefly, a mixture of calcein-AM (C-AM, 1 μmol/L; Sigma-Aldrich) and propidium iodide (PI, 2 μmol/L; Sigma-Aldrich) was prepared. The 3D bioprinted models were washed with PBS and incubated in C-AM/PI mixture for 20 min at room temperature in the dark. After incubation, the 3D bioprinted models were washed three times with PBS and observed under a laser scanning confocal microscope (C2/C2si; Nikon, Tokyo, Japan). Five random fields were captured for each sample, and the cells in the fields were counted using ImageJ (V 1.8.0). Cell viability was calculated by counting the number of cells as follows: Cell viability (%) = (live cells/total cells) × 100%.

### Cell proliferation assay

The 3D bioprinted cells and 2D cells were incubated in a mixture of culture medium and Cell Counting Kit 8 (CCK8; Dojindo, Kumamoto, Japan) at a volume ratio of 9:1. After 2 h of incubation at 37°C, the absorbance of the culture medium at 450/620 nm was measured (AMR-100; ALLSHENG, Hangzhou, Zhejiang, China). The relative rate of cell proliferation was calculated using a standard protocol.

### mRNA expression

The expression of related genes was evaluated using quantitative real-time polymerase chain reaction (qRT-PCR). Total RNA was isolated from 2D RBE, RBE(3D), RBE(TEC), RBE(TAF), and RBE(TAM) using an RNA-quick purification kit (YISHAN Bio Co., LTD, Shanghai, China), according to the manufacturer’s instructions. RNA was reverse transcribed into cDNA using cDNA Synthesis SuperMix (YEASEN Bio Co., LTD, Shanghai, China). Ki-67, OCT-4, EPCAM, MRP2, BCRP, β-catenin, cyclin D1, c-Myc, N-cadherin, and MMP9 mRNA levels were determined by performing qRT-PCR using BlasTaq™ 2X qPCR MasterMix (abm, San Diego, CA, USA) in a real-time PCR system (Applied Biosystems, Foster City, CA, USA). The amplification conditions were as follows: initial denaturation for 30 s at 95°C; 40 cycles of denaturation for 5 s at 95°C, annealing for 30 s at 60°C, and elongation for 30 s at 72°C; and a final extension step for 30 s at 72°C. qRT-PCR was performed in triplicate and the fold change (2^-ΔΔCt^) in the expression of each gene was calculated for each group. Primer sequences are listed in **Supplementary Table 1**.

### Immunofluorescence assay

After washing with PBS, 2D cells and 3D bioprinted cells were fixed in 4% paraformaldehyde (Solarbio) for 20 min. The cells were incubated with 0.1% Triton X-100 (Sigma-Aldrich) for 30 min and 3% bovine serum albumin (BSA; Thermo Fisher Scientific, Waltham, MA, USA) for 45 min. All the aforementioned steps were conducted at room temperature. Then the cells were incubated with primary antibodies for Ki-67 (1:250, Abcam, Cambridge, MA, USA), MRP2 (1:250, Abcam), β-catenin (Cell Signaling Technology, CST, Danvers, MA, USA), and E-cadherin (1:250, Abcam), vimentin (1:250, Abcam) overnight at 4°C, separately. After that, the cells were incubated with fluorochrome-conjugated secondary antibody (1:500, Abcam) and 4′,6-diamidino-2-phenylindole (DAPI, 1:1000, Abcam). Images were obtained using a fluorescence microscope (Nikon).

### Pharmacodynamic evaluation of antitumor drugs

After 7 days of culture, 3D bioprinted cells were treated with different concentrations of gemcitabine (Sigma-Aldrich) (0.01, 0.08, 0.4, 2, 10, and 50 μM), cis-platinum (Sigma-Aldrich) (0.01, 0.08, 0.8, 4, 20, and 100 μM), or 5-fluorouracil (Sigma-Aldrich) (0.01, 0.08, 0.8, 8, 40, and 200 μM) for 72 h. When 2D RBE were 70% confluent, generally 2~3 days after passage, the cells were treated under the same conditions as described above. Cell growth was measured using the CCK8 assay, and dose-response curves were drawn using GraphPad Prism (Version 9.0.0, San Diego, CA, USA).

### Statistical analysis

Data are expressed as the mean ± standard deviation. Statistically significant differences between the groups were determined using Student’s t-test. A significance level of 5% (*p* < 0.05) was used for all tests.

## Results

### Construction of 3D bioprinted model to simulate tumor immune microenvironment

To simulate and recapitulate the *in vivo* growth status of CCA in an *in vitro* environment, we applied 3D bioprinting technology to establish a 3D printed microtissue model (**Figure 1B**) and the model RBE cell printed alone as control (**Figure 1A**). **Figure 1B** shows a schematic of the construction of the 3D CCA immune microenvironment. Hydrogel GelMA was assembled into a grid-like stereo structure with defined pores, which encapsulated the RBE or TEC/TAF/TAM. The single-cell length, width, and height of the model were measured to be approximately 5, 5, and 1 mm, respectively. The outside diameter, inner diameter, and height of the model were 16, 8, and 1 mm, respectively. The ratio of cell number was 1:1.5 (RBE: stromal cells), calculated by hydrogel volume with the same density of cells.

**Figure 2:**
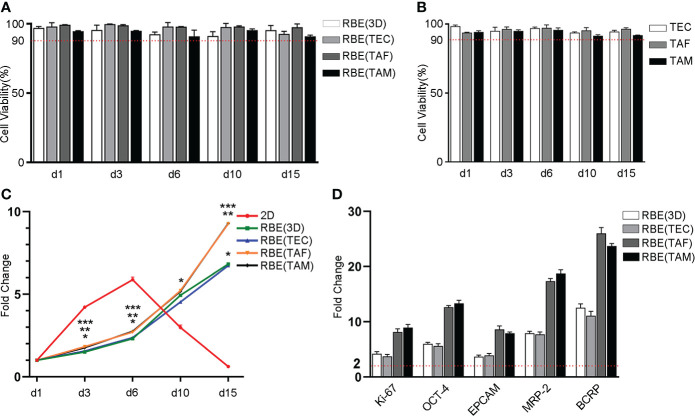
Statistical results of cell viability, proliferation and genes expression in different models. **(A)** The cells viability of RBE was stable above 90% in different 3D bioprinted models throughout the growth cycle. **(B)** Statistical result of stromal cells viability in 3D bioprinting hydrogel, which was all more than 90% in the total growth cycle. **(C)** Statistical result of cell proliferation with CCK8 assay. The result was shown as fold change compared to day 1, respectively. *, 2D vs. RBE(3D), *p* < 0.05. **, RBE(3D) vs. RBE(TAF), *p* < 0.05. ***, RBE(3D) vs. RBE(TAM), *p* < 0.05. **(D)** Statistical result of gene expression by qRT-PCR assay. The result was shown as fold change compared to 2D-RBE, separately. The experiments were replicated at least three times.

C-AM/PI staining was used to determine the cell viability. In the present study, the viability of the 3D bioprinted RBE and stromal cells was stable above 90% during the entire growth cycle (**Figures 2A, B**, **S1**, **S2**). These results indicated that the cells were well grown in the 3D bioprinting environment and did not need to be passaged during the 15-day study period, which better mimics the tumor microenvironment *in vivo*. These findings will help in understanding the real role of stromal cells in tumor cells.

### 3D bioprinted cells have better and stable proliferation capacity

To track the growth of tumor cells over time, we compared the proliferation of 3D bioprinted and 2D cultured RBE using the CCK8 assay. The results were shown as multiples of day 3, 6, 10, and 15 after 3D bioprinting, compared to the data on day 1 (**Figure 2C**). Compared to that of the cells cultured in 2D environment, the growth rate of 3D bioprinted cells was slower after 1 ~ 3 days. However, after 3 days, proliferation rates were similar in all types of environments. In addition, after 6 days, as the 2D cells did not have enough space to grow, the rate of apoptosis began to increase gradually, and the proliferation rate of 3D bioprinted cells was higher than that of the 2D models. In addition, we cannot neglect that RBE co-cultured with TAF or TAM had higher proliferation than those cultured alone or with TEC after 10 days. These results showed that 3D bioprinted cells have improved and more stable proliferation than 2D cells, and the existence of TAF or TAM can promote the proliferation of tumor cells, while TEC did not exhibit this function.

Therefore, we selected 7 days after bioprinting as the time point for functional examination, including gene expression and drug testing, as this time point was considered suitable for comparison of 3D bioprinted cells with planar cultured cells. While 2D cells with 70% confluent and good viability were selected for the above functional examination, normally 2~3 days after passage.

### 3D bioprinted microenvironment promotes tumor-related mRNA and protein expression

**Figure 3:**
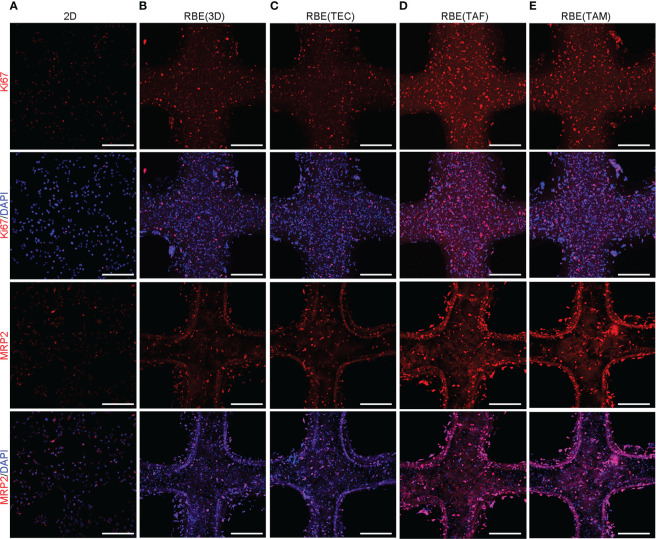
Correlative protein expression by immunofluorescence (IF) in different models. **(A–E)** The expression of Ki67, MRP2 at 7 days after 3D bioprinting and 2D cells with appropriate density. Compared with 2D environment, 3D bioprinted environment promoted genes expression. While RBE(TAF) and RBE(TAM) had higher expression than that in RBE(3D) and RBE(TEC). Scale bars: 40 μm. The experiments were replicated at least three times.

To detect the gene expression levels of cells under different growth conditions, we used quantitative reverse transcription-polymerase chain reaction (qRT-PCR) and immunofluorescence (IF) to measure the relative levels of gene expression after 7 days of culture. The results are shown in **Figures 2D**, **3**. The qRT-PCR results were consistent with those of the IF assays. The gene expression levels of cells cultured in 3D bioprinted models in proliferation, cancer stem cell heterogeneity, and drug resistance were at least 2-fold higher than those of cells cultured in 2D environment (**Figure 2D**). 3D bioprinting conditions can facilitate cell multiplication. In addition, 3D bioprinted cells showed higher resistance to antitumor drugs. Moreover, the presence of stromal cells TAF, and TAM can boost the malignancy of RBE, whereas TEC does not have an effect on RBE.

### Effects of antitumor drugs on the 3D bioprinted models

**Figure 4:**
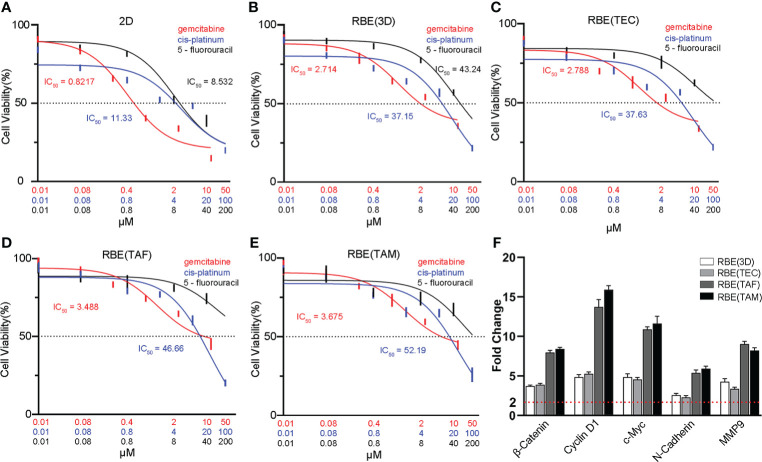
Characterization of drug metabolism and genes expression in different models. Dose-response curves and half maximal inhibitory concentration (IC50) of gemcitabine, cis-platinum and 5-fluorouracil in 2D cell model**(A)**, the RBE(3D) **(B)**, RBE(TEC) **(C)**, RBE(TAF) **(D)** and RBE(TAM) **(E)** after 72 h of incubation. Compared with 2D RBE cells, cells in 3D bioprinted models had higher IC50 values, especially in RBE(TAF) and RBE(TAM). **(F)** qRT-PCR assays showed that 3D bioprinting upregulated the expression of Wnt/β-catenin pathway-related genes (β-Catenin, Cyclin D1 and c-Myc) and epithelial-mesenchymal transition (EMT) markers (N-Cadherin and MMP9), compared with 2D RBE cells. The experiments were replicated at least three times.

To assess the response of cells to antitumor drugs in different models, 3D bioprinted cells and 2D RBE were treated with different concentrations of gemcitabine (0.01, 0.08, 0.4, 2, 10, 50μM), cis-platinum (0.01, 0.08, 0.8, 4, 20, 100μM), and 5-fluorouracil (0.01, 0.08, 0.8, 8, 40, 200μM) for 72 h, separately. The half-maximal inhibitory concentrations (IC50) of gemcitabine in the five groups (2D, RBE(3D), RBE(TEC), RBE(TAF), RBE(TAM)) were 0.8217, 2.714, 2.788, 3.488, and 3.675 μM, respectively. The IC50 values of cis-platinum in the above five groups were 11.33, 37.15, 37.63, 46.66, and 52.19 μM, respectively. The IC50 values of gemcitabine and cis-platinum in the 3D bioprinted models were significantly higher than those in the 2D cells (**Figures 4A–E**) (*p* < 0.05). It was worth noting that in the drug inhibition experiment, the different concentrations of 5- fluorouracil had better inhibitory effects on 2D RBE, RBE (3D), and RBE(TEC) group, while in the 3D bioprinting model containing TAF or TAM cells, high concentrations of 5- fluorouracil were still challenging to show cell inhibition. Overall, the stromal cells TAF and TAM accelerated the resistance against the drugs mentioned above (*p* < 0.05).

### 3D bioprinting drives the process of epithelial-mesenchymal transition may by activating Wnt/β-catenin signaling in CCA

**Figure 5:**
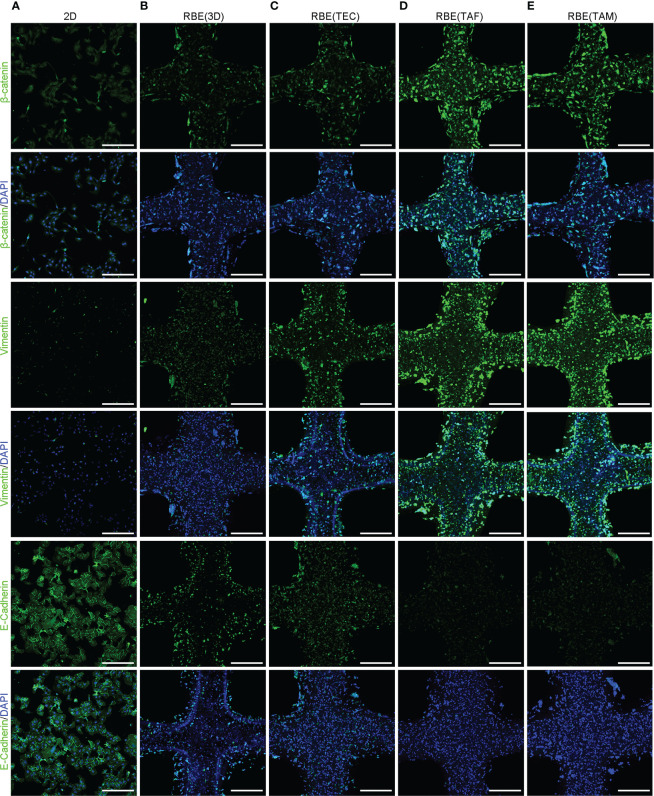
3D bioprinting promotes EMT may *via* the Wnt/β-catenin pathway in CCA. **(A–E)** The results of IF staining, including β-Catenin, Vimentin and E-Cadherin in the 2D at appropriate density and 3D bioprinted models at 7 days after 3D bioprinting. Scale bars: 40 μm. The experiments were replicated at least three times.

To investigate changes in epithelial-mesenchymal transition (EMT) in CCA, qRT-PCR and IF assays were conducted. The results showed that 3D bioprinting significantly promoted the migration and invasion of RBE, increased the expression of vimentin, N-cadherin, and MMP9, and decreased the expression of E-cadherin (**Figures 4F**, **5**). These results suggested that 3D bioprinting is involved in the EMT of CCA cells. To reveal the molecular mechanism of 3D bioprinting in promoting the EMT process, we subsequently performed qRT-PCR and IF to identify the target genes differentially expressed between 3D bioprinted cells and 2D cells. The 3D bioprinted cells showed increased expression of the Wnt/β-catenin pathway genes (β-catenin, cyclin D1, and c-Myc). These results suggest that 3D bioprinting may facilitate the mesenchymal phenotype of tumor cells in a Wnt/β-catenin signaling-dependent manner.

## Discussion

The lack of tumor-related stromal cells in traditional 2D cell cultures is not suitable for studying the function of cells in the tumor environment, which makes it difficult to simulate the real tumor immune environment. Accurate models of tumors are needed to understand how complex stromal cells contribute to tumor growth, progression, and drug response. Associated mouse models and xenografts of cancer cells into immunocompromised mice have benefited the research of tumors, such as the presence of a tumor immune microenvironment. However, these models have shortcomings, such as high costs, time consumption, lack of human stroma, and difficulty in manipulation (21, 43). Hence, the results obtained in animals are not always confirmed in humans (44).

Therefore, establishing an *in vitro* biomimetic tumor model can overcome some of the aforementioned shortcomings. 3D bioprinting technology offers the unique ability to create architecturally and compositionally complex biomimetic microenvironments with high reproducibility. A 3D bioprinted tissue model recapitulates cell-matrix interactions and tumor cell-stromal cell communications, with tissue heterogeneity incorporated into the model (29, 45). Here, an extrusion-based bioprinting strategy was used to fabricate a 3D tumor model to analyze and compare the biological behavior and different roles of stromal cells in the tumor immune microenvironment (46–48). Hydrogels such as GelMA have been widely used for 3D bioprinting many types of models because of their hydrophilic polymeric networks and ECM-like properties (49). Studies have shown that hydrogel-based tumor models with 3D microenvironments and physicochemical properties are critical for studying the interactions between cells and the tumor microenvironment (50). In this study, GelMA was bioprinted because of its shear-thinning property, and after crosslinking, the printed scaffold provided a good mechanical strength (30). Owing to this physicochemical property, the printed linear hydrogel presented a well-defined interconnected channels between two adjacent hydrogels, which ensures a timely and adequate exchange of gases and nutrients between the environment and hydrogel. Many reports have already revealed the advantages of 3D bioprinting in tumor biology (51). However, only few studies have examined the biological functions of tumor-related stromal cells using 3D bioprinting technology. In particular, for CCA, a malignant tumor with abundant stroma, the effects of stromal cells on 3D bioprinted tumor cells have not been reported.

Here, an extrusion-based 3D bioprinting strategy was used to fabricate 3D tumor models to analyze and compare the biological behavior of TEC, TAF, and TAM in 3D immune microenvironments, particularly their different roles in tumor progression. The viability of RBE and stromal cells in different models at different days after bioprinting was always greater than 90%. This suggests that regardless of the cell type, the 3D bioprinting system had good printability and biocompatibility. In addition, the 2D cells have to be passed every few days, whereas the cells in a 3D bioprinted system can be cultured for more than 15 days continuously, which can be used for long-term experiments. In the present study, the CCK8 assay, Ki-67 testing with qRT-PCR, and IF assays were conducted to detect the proliferation of cells in different models. Although 2D cells may have higher proliferation than those of 3D bioprinted models in 1 ~ 6 days, 2D cells began to die after 6 days because of waste accumulation and lack of growth space. Throughout the course of the study, the proliferation and growth of RBE in the 3D bioprinted models were higher than those in the 2D environment.

Studies have indicated that cancer stem cells (CSCs), a small subpopulation of cancer cells with tumor-initiating capabilities, are responsible for tumorigenesis (52). It is widely believed that CSCs are closely related to pathological features, such as worse clinical prognosis. The presence of CSCs in a tumor is closely related to enhanced invasiveness and metastatic capability (53, 54). Resistance to conventional anti-cancer drugs is a characteristic of CSCs (55, 56). In addition, CSCs can differentiate into phenotypically varied subclones, thereby increasing their resistance to anti-cancer drugs (57). Earlier studies have demonstrated that microenvironments include hypoxic environment, which promotes the generation and maintenance of CSCs (58). However, the grid-like structure of 3D bioprinted model and good permeability of GelMA allows cells in model had adequate and timely oxygen and nutrient exchanges. Therefore, the high proliferative state of CSCs cannot be attributed to hypoxia but to the 3D structure provided by the 3D bioprinting technology. The expression of stem cell markers, OCT-4, and EPCAM in the 3D bioprinting environment was shown to be stronger than that in the 2D environment. The expression of the drug-resistance-related genes, *MRP2* and *BCRP*, was consistent with that of stem cell markers. We found that the IC50 values of the three tested drugs were much higher in the 3D bioprinted models than in the 2D models.

The enhancement of cell malignancy in a 3D bioprinted environment also reflects changes in the expression of proteins related to the EMT process. We investigated the expression of N-cadherin, E-cadherin, vimentin, and MMP9, and found that the epithelial characteristics of tumor cells in the 3D bioprinted environment were decreased, whereas the mesenchymal characteristics increased. This indicates that the 3D bioprinted environment promotes cell malignancy and is conducive to the expression of the tumorigenic phenotype of tumor cells.

The classical Wnt/β-catenin pathway is a complex, evolutionarily conserved signaling mechanism that regulates fundamental physiological and pathological processes (59). It tightly controls embryogenesis, including hepatobiliary development, maturation, and zonation (60). In the mature healthy liver, the Wnt/β-catenin pathway is usually inactive and can be re-activated during cell renewal and regenerative processes, and in certain pathological conditions, diseases, and cancer (61). Normal activation of Wnt/β-catenin signaling is highly prevalent in CCA tumors and can promote tumor cell proliferation and survival (62, 63). Our study showed that 3D bioprinting models exhibited higher expression of genes related to the Wnt/β-catenin pathway, including β-catenin, cyclin D1, and c-Myc, as well as the downstream genes, such as those encoding for N-cadherin, vimentin, and MMP9. 3D bioprinting promotes the EMT of RBE *via* the Wnt/β-catenin pathway by changing the microenvironment. These results provide reliable evidence to reveal the mechanism by which 3D bioprinting models promote tumor cell invasion and metastasis.

Stromal cells in the tumor immune microenvironment are important for disease progression and drug responses. We fabricated *in vitro* 3D 3Dellular CCA models with activated stromal cells to provide a microenvironment that simulates the metabolism and physiological characteristics of *in vivo* tumor tissues to study the function of stromal cells. As the tumor immune microenvironment plays a vital role in tumor progression, we incorporated TEC, TAM, and TAF separately into our experiments, which have been shown to play important roles in tumor progression in other tumors or models (64). Considering that traditional research models may lead to a bias in the results, for the first time, we used 3D bioprinting technology to study the roles of various stromal cells in CCA. The results showed that compared to that in case of 3D bioprinted RBE alone and 3D bioprinted RBE with TEC, TAM and TAF promoted RBE viability. This is embodied in the following aspects. First, TAM and TAF accelerated cell proliferation. Second, TAM and TAF facilitated CSC expression. Third, TAM and TAF enhanced the expression of drug-resistance genes as well as IC50 values. Fourth, the Wnt/β-catenin pathway was extensively activated in 3D bioprinted models of RBE using TAM or TAF. These results align with findings from previous studies.

A previous study shown that TEC induces tumor progression and invasion by activating a series of pathways (65). In addition, TEC can release inflammatory chemokines to attract leukocytes to establish a pro-fibrotic and pro-angiogenic microenvironment and promote migration, invasion, and EMT in CCA (66). Besides, CCA-associated endothelial cells highly express the erythropoietin receptor, which binds to cell-released erythropoietin in the tumor immune microenvironment, thus promoting proliferation, survival, and invasion of CCA cells (67). However, reports have claimed that endostatin precursors secreted by CCA stromal endothelial cells suppress tumor angiogenesis, growth, and development. Weakening of the adhesion between CCA cells and vascular endothelial cells results in the suppression of CCA cell metastasis.

Functional TEC affects the physiological characteristics and progression of CCA cells. However, our results showed that the function of RBE in the RBE(TEC) was not affected, and the results were the same as those in case of RBE(3D), but significantly different from those in case of RBE(TAF) and RBE(TAM). Our analysis suggests that the functional expression of TEC was established based on the premise that it was a part of the tumor vessels. However, TEC distribution in the hydrogel was uniform and did not form a capillary network; thus, it could not exhibit the macro-function of vessels, nutrition/waste products, and gas exchange. In addition, the diameter of our hydrogel was 0.25 mm and the TEC were cultured separately. Therefore, TEC do not play a significant role in this model.

We developed *in vitro* 3D bioprinted tumor model to explore the role of stromal cells in CCA. The results showed that cells in our models can survive for an extended period post-printing, wherein they can be continuously monitored over two weeks without passaging. Also, that the 3D bioprinted model significantly promoted the proliferation and gene expression of tumor cells compared to the 2D and RBE(3D) models. Tumorigenic phenotypes, including the degree of malignancy, stemness, invasiveness, and metastatic ability, were observed to be highly upregulated in RBE(TAF) and RBE(TAM) compared to those in 2D cultures and other 3D models. 3D bioprinting models simulate the tumor microenvironment so that the heterogeneity of the tumor cells and the characteristics of CSCs can be mimicked. Anti-cancer drug resistance of RBE in the abovementioned models further demonstrated their stemness-like properties. In addition, the 3D bioprinted model acts on the tumor cell microenvironment and promotes RBE cells may through the Wnt/β-catenin pathway. Cells in a 3D bioprinted model have a greater ability to invade and metastasize. In conclusion, these 3D bioprinted models exhibit promising potential in the research on tumor progression with a focus on the immune microenvironment and personalized therapy. The established 3D bioprinting route is also applicable and is expected to be used for engineering other tumor tissue models *in vitro*.

## Data availability statement

The original contributions presented in the study are included in the article/**Supplementary Material**. Further inquiries can be directed to the corresponding authors.

## Author contributions

Conceived and designed the experiments: HY, YM, XS, and CL. Performed the experiments: CL, BJ, and HS. Analyzed the data: CL, YW, HZ, XS, and HY. Contributed reagents/materials/analysis tools: HY, YM, BJ, and HS. Wrote the manuscript: CL, BJ, and HS. All authors contributed to the article and approved the submitted version.

## Funding

This work was supported by grants from Beijing Natural Science Foundation (7212077), CAMS Innovation Fund for Medical Sciences (CIFMS) (No.2021-I2M-1-058) and Tsinghua University-Peking Union Medical College Hospital Cooperation Project (PTQH201904552).

## Conflict of interest

The authors declare that the research was conducted in the absence of any commercial or financial relationships that could be construed as a potential conflict of interest.

## Publisher’s note

All claims expressed in this article are solely those of the authors and do not necessarily represent those of their affiliated organizations, or those of the publisher, the editors and the reviewers. Any product that may be evaluated in this article, or claim that may be made by its manufacturer, is not guaranteed or endorsed by the publisher.
