# Supplementary figures and images for: Exploring the function of stromal cells in cholangiocarcinoma by three-dimensional bioprinting immune microenvironment model
Source: Front Immunol. 2022 Aug 2;13:941289. doi: 10.3389/fimmu.2022.941289 (PMC9378822; doi:10.3389/fimmu.2022.941289)

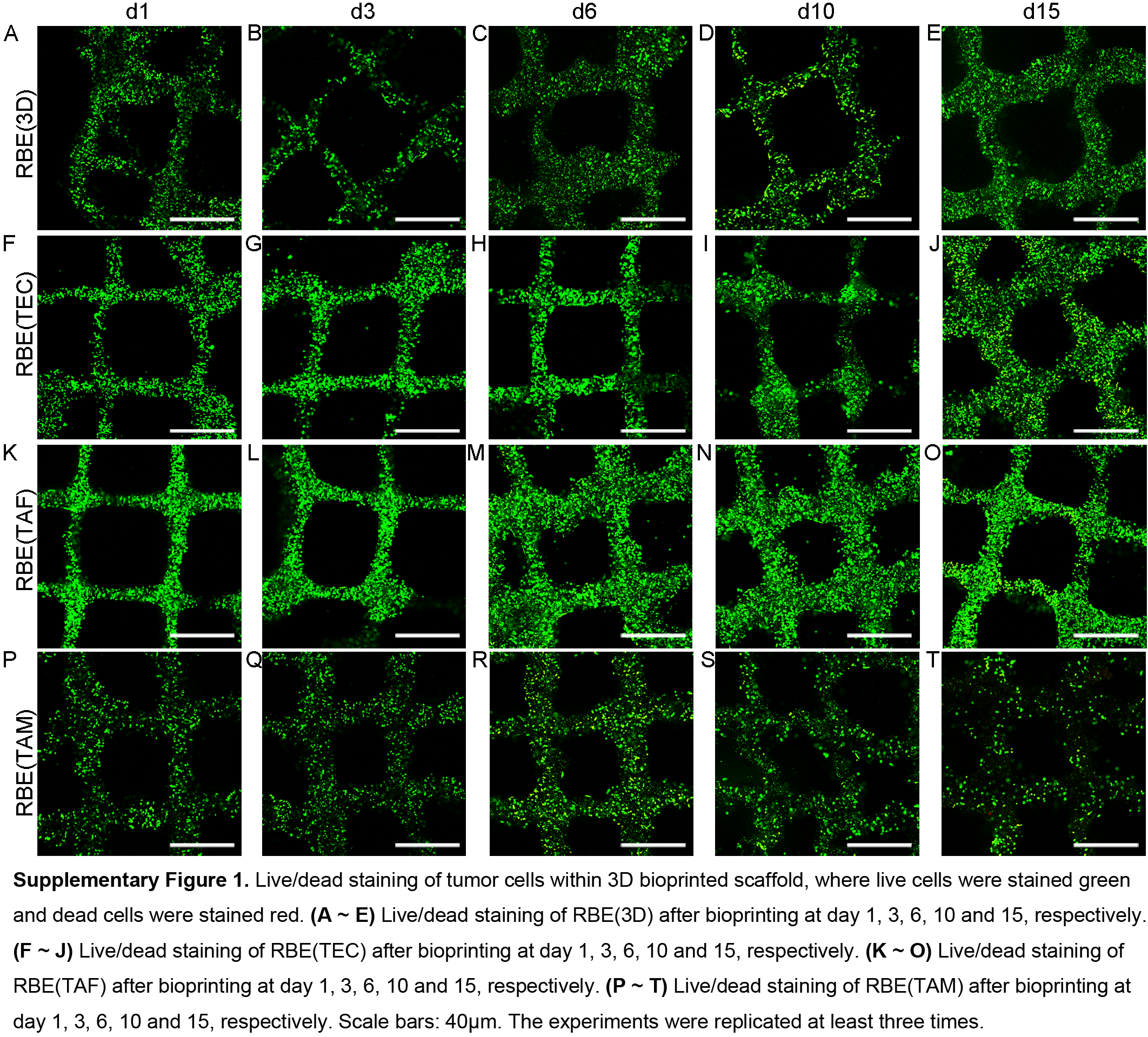

Supplement: Supplementary file 1 [file Image_1.tif]

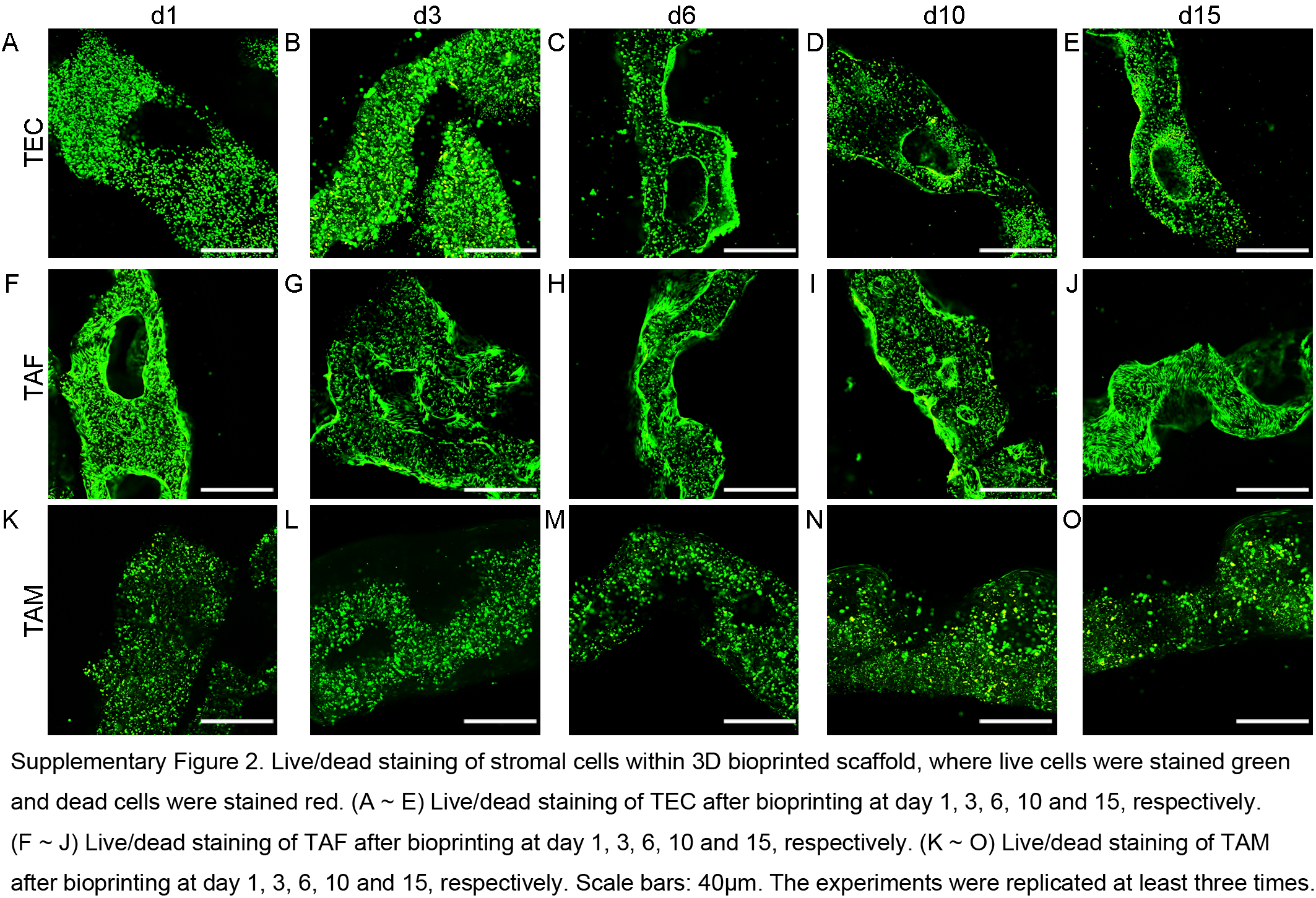

Supplement: Supplementary file 2 [file Image_2.tif]
