# Supplementary material for: Exploring the function of stromal cells in cholangiocarcinoma by three-dimensional bioprinting immune microenvironment model
Source: Front Immunol. 2022 Aug 2;13:941289. doi: 10.3389/fimmu.2022.941289 (PMC9378822; doi:10.3389/fimmu.2022.941289)
Supplement: Supplementary file 3 [file Table_1.docx]

**Supplementary** **Table 1 The sequences of primers used for qRT-PCR**

| **Gene** | **Forward Primer** | **Reverse Primer** |
| --- | --- | --- |
| Ki-67 | CTTTGGGTGCGACTTGACG | GTCGACCCCGCTCCTTTT |
| OCT-4 | GAAGCAGAAGAGGATCACCTTG | TTCTTAAGGCTGAGCTGCAAG |
| EPCAM | AATCGTCAATGCCAGTGTACTT | TCTCATCGCAGTCAGGATCATAA |
| MRP2 | CCCTGCTGTTCGATATACCAATC | TCGAGAGAATCCAGAATAGGGAC |
| BCRP | GTTTCAGCCGTGGAAC | CTGCCTTTGGCTTCAAT |
| β-Catenin | AAAGCGGCTGTTAGTCACTGG | CGAGTCATTGCATACTGTCCAT |
| Cyclin D1 | CTTCAAATGTGTGCAGAAGGAGG | GCTCTTTTTCACGGGCTCCA |
| c-Myc | GGCTCCTGGCAAAAGGTCA | CTGCGTAGTTGTGCTGATGT |
| N-Cadherin | TCAGGCGTCTGTAGAGGCTT | ATGCACATCCTTCGATAAGACTG |
| MMP9 | TGTACCGCTATGGTTACACTCG | GGCAGGGACAGTTGCTTCT |
| β-actin | CATGTACGTTGCTATCCAGGC | CTCCTTAATGTCACGCACGAT |
